# Motifome comparison between modern human, Neanderthal and Denisovan

**DOI:** 10.1186/s12864-018-4710-1

**Published:** 2018-06-18

**Authors:** Matyas F. Cserhati, Mary-Ellen Mooter, Lauren Peterson, Benjamin Wicks, Peng Xiao, Mark Pauley, Chittibabu Guda

**Affiliations:** 1 Department of Genetics, Cell Biology and Anatomy, University of Nebraska Medical Center, College of Medicine, Omaha, NE 68198-5145 USA; 2 Information Technology Services, University of Nebraska Medical Center, Omaha, NE 68198-5030 USA; 3 School of Interdisciplinary Informatics, University of Nebraska at Omaha, Omaha, NE 68182-0116 USA; 4 Buildertrend Solutions, Inc, Omaha, NE 68154 USA

**Keywords:** Human, Neanderthal, Denisovan, Genome, Promoter, UTR, Intron, Motifome

## Abstract

**Background:**

The availability of the genomes of two archaic humans, Neanderthal and Denisovan, and that of modern humans provides researchers an opportunity to investigate genetic differences between these three subspecies on a genome-wide scale. Here we describe an algorithm that predicts statistically significant motifs based on the difference between a given motif’s actual and expected distributions. The algorithm was previously applied to plants but was modified for this work.

**Results:**

The result of applying the algorithm to the human, Neanderthal, and Denisovan genomes is a catalog of potential regulatory motifs in these three human subspecies. We examined the distributions of these motifs in genetic elements including human retroviruses, human accelerated regions, and human accelerated conserved noncoding sequences regions. Differences in these distributions could be the origin of differences in phenotype between the three subspecies. Twenty significant motifs common to all three genomes were found; thirty-three were found in endogenous retroviruses in Neanderthal and Denisovan. Ten of these motifs mapped to the 22 bp core of MiR-1304. The core of this genetic element regulates the ENAM and AMTN genes, which take part in odontogenesis and whose 3’ UTRs contained significant motifs. The introns of 20 genes were found to contain a large number of significant motifs, which were also overrepresented in 49 human accelerated regions. These genes include NAV2, SorCS2, TRAPPC9, GRID1, PRDM16, CAMTA1, and ASIC which are all involved in neuroregulation. Further analysis of these genes using the GO database indicates that many are associated with neurodevelopment. Also, varying numbers of significant motifs were found to occur in regions of the Neanderthal and Denisovan genomes that are missing from the human genome, suggesting further functional differences between modern and archaic humans.

**Conclusion:**

Although Neanderthal and Denisovan are now extinct, detailed examination of elements from their genomes can shed light on possible phenotypic and cognitive differences between these two archaic human subspecies and modern humans. Genetic similarities and differences between these three subspecies and other fossil hominids would also be of interest.

**Electronic supplementary material:**

The online version of this article (10.1186/s12864-018-4710-1) contains supplementary material, which is available to authorized users.

## Background

The recent sequencing of the Neanderthal and Denisovan genomes has provided an exciting opportunity to unravel the genetic differences between modern humans and our two closest relatives [1–3]. Up until now, the majority of analyses performed on the Neanderthal and Denisovan genomes have been restricted to the analysis of polymorphisms, population dynamics, and individual genes; little has been done with respect to analyzing genetic regulation. Since the two archaic hominin subspecies are extinct, such a study is made difficult by the fact that direct examination of gene activity is not possible. However, as the genomes of modern human, Neanderthal, and Denisovan (HND) are very similar to each other—some have theorized that modern humans, Neanderthals and Denisovans interbred [4]—gene activity can be inferred by examining changes in promoters and other regulatory regions which, in turn, would correspond to changes in transcription factor binding sites [5]. Thus, the presence or absence of motifs in the promoter regions of these subspecies could indicate biological, and therefore phenotypic, differences between them and could shed light on the molecular basis of human genetic variation [6–9].

Armed with the HND genomes, a detailed genomic analysis of these three subspecies can be performed. Here we are interested in differences in the motif content of genes. Indeed, several genes have been identified which exhibit variation between modern humans and Neanderthals. These include the ABO blood group locus, a taste receptor, as well as the gene MC1R which could code for red hair and light skin [10]. In addition, a number of genetic elements—e.g., human accelerated regions (HARs), human accelerated conserved noncoding sequences regions (HACNSs), and transposon elements, such as microRNAs (miRNA) elements and endogenous retroviruses—have been discovered that reflect functional differences between modern humans and the archaic hominins. HACNSs are important in that they are uniquely conserved sequences (thus indicating function) in human and contain cis-regulatory transcriptional enhancers active during development; transposon and microRNA elements are important since about 40% of the human genome is made up of retrotransposons [11] and since microRNA elements regulate more than 30% of all protein-coding genes [12].

In this paper, we use an algorithm described in previous works [13, 14] to generate and rank a catalogue of all motifs in the whole genomes and several sub-genomic regions in human, Neanderthal, and Denisovan. In part, the algorithm calculates the difference between a given motif’s actual distribution and its expected distribution based on the base pair content of the genome. We then determine if the significant motifs, those for which the actual occurrence is higher than expected, have any biological significance by looking for them in the high-quality transcription factor binding profile JASPAR database [15] and determining whether they are present in any genetic elements (promoters, miRNAs, functionally conserved non-coding regions, etc.). The presence of these significant motifs could indicate biological differences between modern and archaic humans. Other researchers can use these motifs in their own research to help in the discovery of possible functional genetic elements and to further elucidate the genetic differences between modern and archaic humans.

## Methods

### Sequence sets

For human, the whole genome sequence was downloaded from the RepeatMasker website (SCR_012954) [16]: http://www.repeatmasker.org/genomes/hg19/RepeatMasker-rm330-db20120124/hg19.fa.out.gz. The core, proximal, and distal promoter sets were downloaded from the Eukaryotic Promoter Database (EPD) (SCR_002485) (https://epd.vital-it.ch/seq_download.php) [17]. Core promoters were determined to be 300 bp long, proximal promoters as 1000 bp long, and distal promoters to be 3000 bp long. The EPD database is built on the hg19 genome assembly. The introns for human (build 37.1) were downloaded from http://bpg.utoledo.edu/~afedorov/lab/EID/hs37p1.EID.tar.gz [18], and the human 5′ and 3’ UTR sets were downloaded from http://utrdb.ba.itb.cnr.it/home/download [19]. The human sequence sets were filtered with Repeat Masker [16].

Mouse was selected as an outlier species with which to compare the three hominin subspecies. We chose mouse because it is a well-tested mammalian system, and also has available the corresponding sequence sets with human. The mouse whole genome sequence was downloaded from the UCSC website (SCR_005780): http://hgdownload.cse.ucsc.edu/goldenPath/mm10/bigZips/chromFaMasked.tar.gz. The promoter sequences as well as the 5′ and 3’ UTRs were also downloaded together with the human data sets. The mouse introns (build 37.1) were also downloaded from the EID database: http://bpg.utoledo.edu/~afedorov/lab/EID/mm37p1.EID.tar.gz.

The chimpanzee genome was downloaded from the UCSC website: http://hgdownload.cse.ucsc.edu/goldenPath/panTro2/bigZips/chromFa.tar.gz.

The vcf files for the Neanderthal and Denisovan genome were downloaded from http://cdna.eva.mpg.de/neandertal/altai/AltaiNeandertal/ and http://cdna.eva.mpg.de/denisovan/ and converted to fasta format by a python script. A database was made from these sequences and the human reference transcript was aligned to orthologous Denisovan transcripts to retrieve promoter and intron sequences.

The whole genome sequence of Neanderthal and Denisovan as well as the core, proximal, and distal promoter sets and the set of all introns of Denisovan are available at http://golgi.unmc.edu/HumanMotifomeData/.

The sequence for has-mir-1304 was retrieved from the miRBase database (SCR_003152) [20] at http://www.mirbase.org/cgi-bin/get_seq.pl?acc=MI0006371. The chimp genome (panTro2) was downloaded from http://hgdownload.cse.ucsc.edu/goldenPath/panTro2/bigZips/chromFa.tar.gz. The annotation for human genes was retrieved from the GeneCards database (http://www.genecards.org/).

### Scoring and selection procedure of motifs

The method for predicting and scoring motifs in a given sub-genomic set of sequences builds upon the methods of previous works [13, 14, 21, 22]. The reader is referred to these papers for a detailed description of motif prediction and scoring. In this work, however, we have refined and improved the motif detection algorithm to provide more robust results.

First of all, the sequences used were all filtered for repeat sequences using the RepeatMasker software. Next, the motif scoring scheme was normalized. According to the new method, the motif score is now

$$ S= Obs/\left( Exp+ Obs\right) $$

where *Obs* is the observed occurrence of a given motif within a given sub-genomic set of sequences, and *Exp* is the expected number of occurrences, given the base pair distribution (%A, C, G, and T). This way, the score value will always be between 0 and 1. A score of S = 0.5 means that the motif occurs just as many times as it is expected to occur and is biologically meaningless. Higher scores (closer to 1.0) correspond to motifs which occur more times than expected, and thus correspond to biological relevance. Lower scores (closer to 0.0) correspond to motifs which occur less times than expected, and thus correspond to biological insignificance. This score value is calculated for all combinatorically possible motifs of a given length.

In the second step, the motifs of a given set are ranked in decreasing order according to their score value. For each motif length (k = 6...10), and for each sub-genomic set of sequences, the average score value and the standard deviation are also calculated. A cutoff score value of S_cut_ = S_av_ + 2⋅stdev is calculated. The reason 2 standard deviations are used is because this corresponds to a 5% significance level, according to the normal distribution. Each motif with a score value above the cutoff value was taken to be significant.

**Fig. 1:**
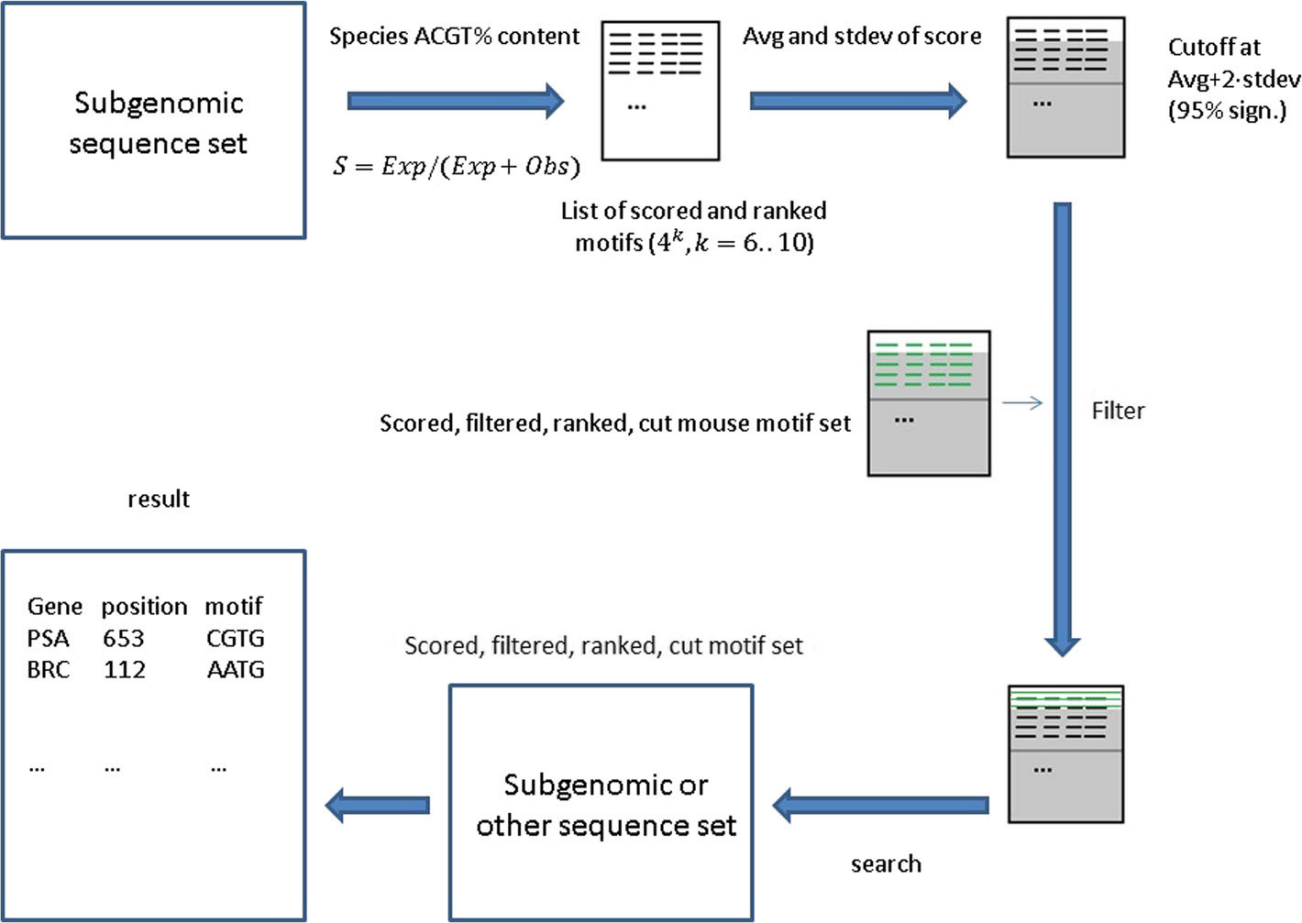
Graphical overview of application of algorithm. First, the A, C, G, T% for a given subgenomic sequence set is determined. Next, the statistics for each motif (for a given motif length from 6 to 10) is generated. This involves the calculation of the actual and expected occurrence for each motif as well as the score for each motif. Next, the set of motifs are filtered whose score is at least two standard deviations above the average score value. Next, the motif set is filtered again to remove general mammalian motifs. For this, the corresponding mouse motif set was used. Lastly, this set of statistically significant, double-filtered motif set can be used to search a given set of subgenomic or other sequence set

In the next step, the same procedure was performed for a set of corresponding motifs from mouse. Mouse was used so as to filter out general mammalian motifs which are not specific to human, Denisovan, or Neanderthal. Thus, we would arrive at a set of significant human sequences and mouse sequences. The set of biologically significant sequences in human were then filtered with the significant mouse sequences. The number of significant motifs, their average score values and standard deviation values are provided in the Additional files 1, 2, 3 and 4 for human, Neanderthal, Denisovan and mouse. This way, for each motif length and each sub-genomic set of repeat-masked sequences, for each of the three hominin subspecies we were able to determine a set of normalized, filtered motifs for each of the sub-genomic regions. The whole process can be seen in Fig. 1. Furthermore, the set of motifs were also validated in the next step by comparing them against the human position weight matrices (PWMs) from the JASPAR database.

The *p*-value for common motifs of lengths 6 to 10 bp for different subgenomic regions between modern human and Denisovan was calculated in Excel using the hypergeometric distribution.

### JASPAR database validation

Position frequency matrixes (PFMs) from the JASPAR database (SCR_003030) [15] and transformed into Position Weight Matrixes (PWMs) for human and mouse. Human PFMs were also used for Neanderthal and Denisovan (due to the similarity of the subspecies). Each motif from each sub-genomic sequence set and the whole genome for all motif lengths from 6 to 10 bp were matched against these PWMs, and the annotation for each such motif was noted. These annotations were marked for all scored motifs, which were ranked by decreasing score values. Each motif was marked with a 1 signifying the presence of at least one matching JASPAR motif and with a 0 if it didn’t match anything. We applied a statistical test where we took the ranks of all matching motifs, and the ranks of all non-matching motifs, and ran a t-test comparing these values with each other. These *p*-values are available for all sequence sets and all motif lengths from 6 to 10. In each and every case the *p*-value was statistically very significant (*p* < 1e-3).

### Motif search

#### Conversion of coordinates of genetic elements

The conversion of the coordinates of the HAR elements (hg17) from Pollard et al. [23] and the conversion of the SNP/INDEL coordinates (hg18) from Zhang et al. [24] were performed at the UCSC site using the liftover utility (https://genome.ucsc.edu/cgi-bin/hgLiftOver) to translate these coordinates to hg19 coordinates.

### Other

**Fig. 2:**
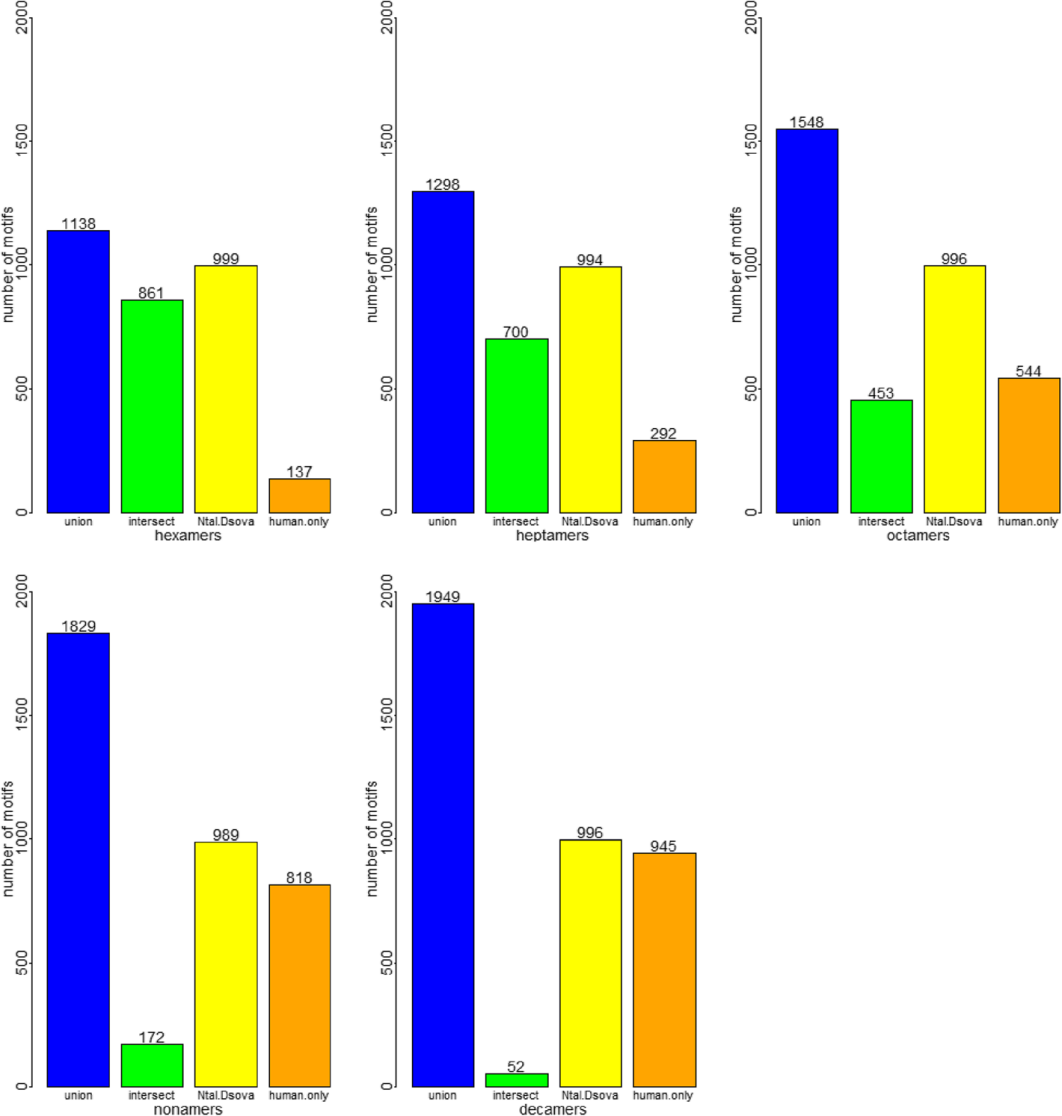
Combination of top 1000 genome motifs from modern human, Neanderthal and Denisovan. The top 1000 genome motifs (hexamers to decamers) from modern human and the two archaic human species were compared with each other. What we can observe is that as the motif length increases, the intersect between all three species decreases, whereas the union of all motifs increases. The number of motifs common to both Neanderthal and Denisovan remain constantly very high, whereas the number of motifs unique to modern humans increases

**Fig. 3:**
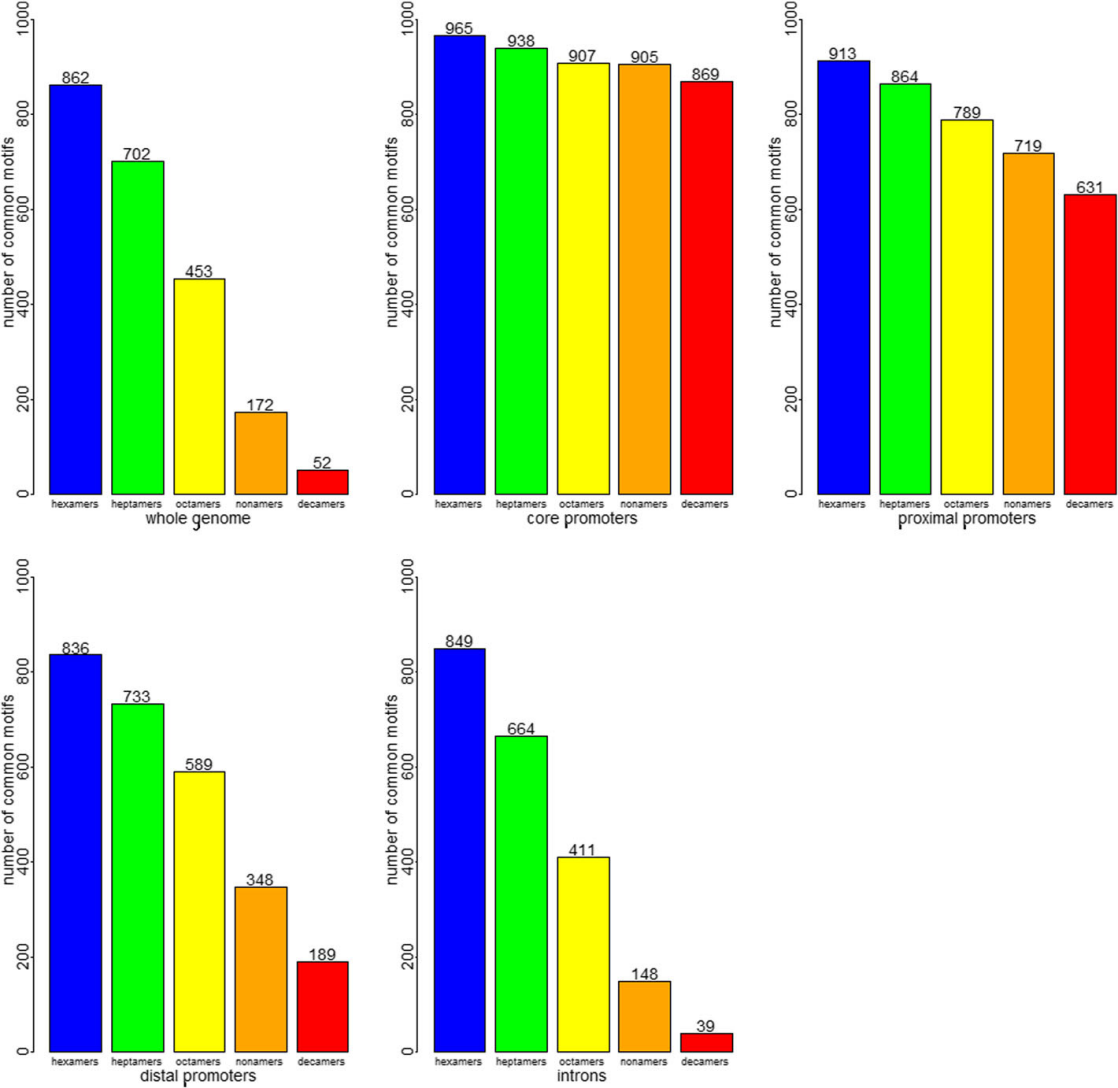
Number of common motifs between modern human and Denisovan from different genomic regions. The top 1000 motifs coming from the whole genome, core, proximal, and distal promoters as well as all introns were compared between modern human and Denisovan for motif lengths 6–10. As we can see, as the length of the motif increases, the smaller number of common motifs. This is due to the fact that the longer the motif gets, the larger the possible number of motifs, thereby making it less likely that two species have the same motif. For core promoters, it is interesting to note that modern human and Denisovan have 869 decamer motifs in common (despite there being 1,048,576 possible decamers). *P*-values were calculated for each genomic sub-region and each motif length, and can be seen in Additional file 5. All *p*-values were extremely statistically significant

**Fig. 4:**
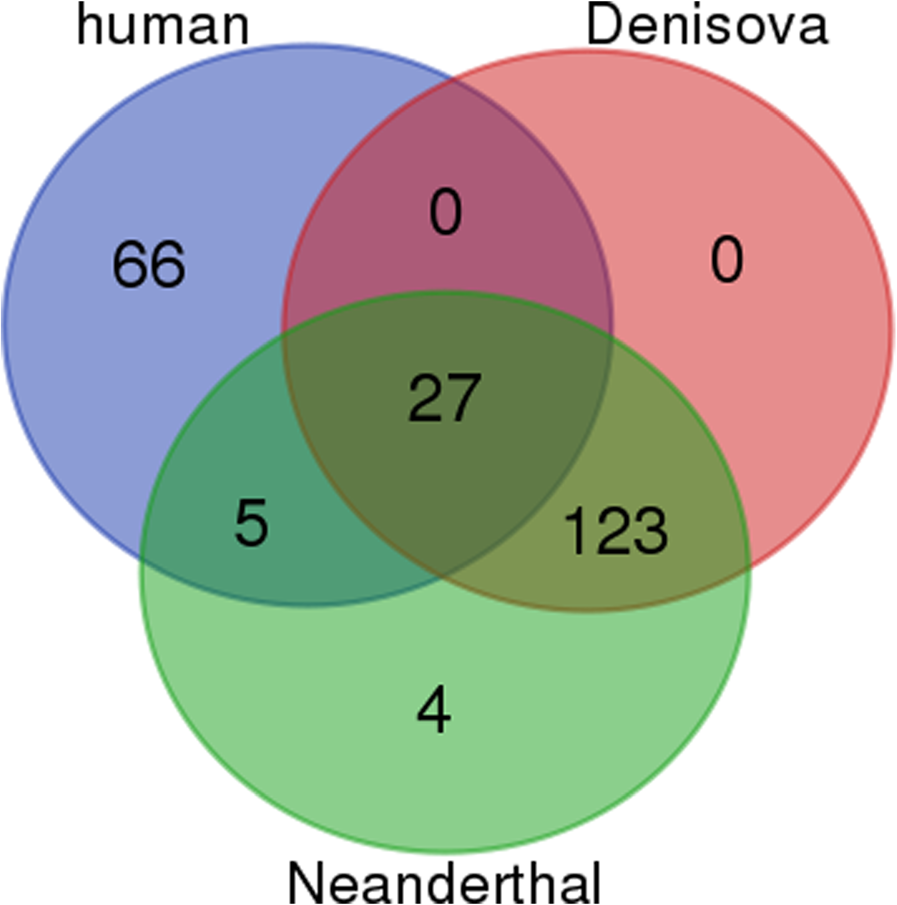
Statistically significant intergenic motifs between modern and archaic humans. The Venn diagram shows the number of statistically significant whole genome motifs common to different combinations of modern human, Neanderthal and Denisovan found in the 26 intergenic HAR regions described by Pollard et al. (2006) [23]

**Fig. 5:**
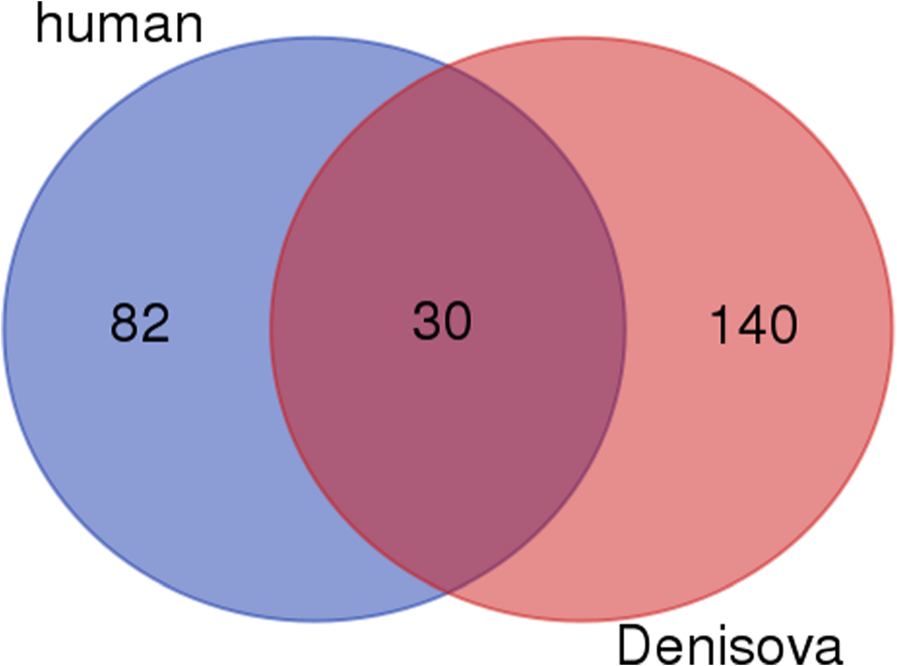
Statistically significant intronic motifs between modern human and Denisovan. The number of statistically significant whole genome motifs common to different combinations of modern human and Denisovan found in the 20 intergenic HAR regions described by Pollard et al. (2006) [23]. The 82 motifs unique to modern humans were then searched for in the intron sequences of modern human. The top 189 transcripts which had the highest number of these motifs were then subsequently mapped to 135 UniProt IDs at the UniProt website, which in turn mapped to 261 specific gene names, for which GO analysis was performed

Transcript IDs were mapped to UniProt IDs at http://www.uniprot.org/uploadlists/ (selecting From RefSeq Protein to UniProtKB) (SCR_002380). Gene Ontology Analysis was performed at the PANTHER website (SCR_004869) [25]: http://pantherdb.org/webservices/go/overrep.jsp. Figures 2 and 3 were made in R, and Figs. 4 and 5 were made using the Venn diagram software at http://bioinformatics.psb.ugent.be/webtools/Venn/ . Chi-squared analysis for testing the statistical significance of the CG% of the three subspecies in Table 2 was performed by using the chisq.test function in R.

## Results

### Principle of investigation

In this paper, we use the term “motif” to mean a short stretch of DNA, 6–10 bp long, which overlaps the core of a transcription factor binding site; the motifome of an organism consists of all combinatorically possible motifs of all possible lengths, 6–10 bp in this case. For example, there are 65,536 (= 4^6^) possible hexamers in the hexamer motifome (all nucleic acid motifs of length six, ranging lexicographically from AAAAAA to TTTTTT). Assuming a random background nucleotide distribution, we would expect that motifs which occur in higher numbers than expected do so because they have experienced positive selective pressure due to the functionality they convey. Using the same reasoning, motifs which occur at the expected frequency or lower are considered biologically irrelevant [26]. The present work defines the motifomes of the three hominin subspecies—human, Neanderthal, and Denisovan—and predicts motifs which may take part in the regulation of gene sets that could cause phenotypic differences between the three subspecies.

Building on and refining the methodology of previous work [13, 14, 22], we measured the statistical significance of the motif content of the whole genomes of human, Neanderthal, and Denisovan (as well as mouse for reference). Motifs in specific regions of the genome—core promoters, proximal promoters, distal promoters, all introns, 5’ UTRs and 3’ UTRs—were also determined so that these regions could be examine separately (see Table 1). Core, proximal, and distal promoters are defined as the segments of DNA 300, 1000, and 3000 bp upstream of the start codon of the coding sequence of a gene. The resulting set of statistically significant motifs was normalized by score and was double-filtered to remove general mammalian motifs (by filtering out those motifs, which also occurred in mouse) as well as low-scoring motifs, those whose actual occurrence is close its expected occurrence. For a detailed description of the algorithm, see [13, 14]. An overview is given in Fig. 1, and is described in more detail in the Methods section. The motifs predicted by the algorithm as being statistically significant (that is, with a much higher occurrence than is expected) for all three subspecies and mouse can be found in the Additional files 1, 2, 3 and 4.

**Table 1:** Genomic regions analyzed in human, Denisovan and Neanderthal

Region	Modern human	Denisovan	Neanderthal
Whole genome	√	√	√
Core promoters	√	√	X
Proximal promoters	√	√	X
Distal promoters	√	√	X
Introns	√	√	X
5’ UTRs	√	X	X
3’ UTRs	√	X	X

### Motif comparison between human, Neanderthal, and Denisovan

#### Whole genome motifs

Only the whole genome sequences of Neanderthal and Denisovan were available (see Methods), so we compared the 1000 highest-scoring significant whole genome motifs from modern human, Neanderthal, and Denisovan. The number of common motifs between different combinations of subspecies can be seen in Fig. 2. The general trend is that as the length of the motifs increases from six to ten, the union of significant motifs in all three subspecies increases, whereas the intersection between all three subspecies decreases. Furthermore, despite this trend, the number of significant motifs common to Neanderthal and Denisovan remains close to 1000, whereas the number of significant human-specific motifs increases steadily from 137 to 945. This might indicate that the whole genome sequences of Neanderthal and Denisovan are very similar (according to Table 2, their ACGT% is almost identical), thus allowing for a greater turnover in motif content. The reason the number of common motifs with human decreases as the length of the motif increases is due to the fact that longer motifs are more specific sequentially, and their relative abundance is lower. A list of the top 20 significant motifs found in all three subspecies’ genomes can be seen in Table 3.

**Table 2:** ACGT% values for the human, Neanderthal and Denisovan genomes

Species	A	T	C	G
Modern human	29.78%	29.82%	20.19%	20.21%
Neanderthal	29.53%	29.57%	20.45%	20.45%
Denisovan	29.52%	29.57%	20.45%	20.46%
Chi-square statistic	1.17 × 10^−6^		4.98 × 10^−6^	
*p*-value	1.0		1.0

**Table 3:** Top 20 significant whole genome motifs found in the genomes of all three species

Motif	Human score	Denisovan score	Neanderthal score
CCTCCC	0.818	0.87	0.87
GGGAGG	0.817	0.869	0.869
CCCAGG	0.825	0.854	0.854
CCTGGG	0.825	0.854	0.854
CCCAGC	0.797	0.859	0.859
GCTGGG	0.797	0.859	0.859
CACACACACA	0.978	0.996	0.996
CCAGGC	0.787	0.848	0.847
CCCAGGC	0.856	0.916	0.916
GCCTGG	0.787	0.847	0.847
GCCTGGG	0.856	0.915	0.915
ACACACACAC	0.976	0.996	0.996
CCAGCC	0.789	0.844	0.844
CACACACAC	0.95	0.988	0.988
GGCTGG	0.789	0.844	0.844
CCTGCC	0.802	0.821	0.821
GGCAGG	0.802	0.82	0.82
GTGTGTGTG	0.95	0.988	0.988
AAAAAAAAA	0.949	0.987	0.987
AAAAAAAA	0.899	0.965	0.966

The motif sequence and score are listed for modern human, Neanderthal and Denisovan

#### Motif comparison between human and Denisovan in different sub-genomic regions

Significant motif content was compared between human and Denisovan. The number of common motifs between modern human and Denisovan in the whole genome, the core, proximal, and distal promoters and all introns can be seen in Fig. 3. Here we can also see that with increasing motif length, the number of common motifs decreases. The decrease is steepest for the whole genome and for introns, and the least steep for core and proximal promoters. This means that even though there might be differences in the genome sequence between modern and archaic humans, the regulatory regions have few differences and have not diverged from each other. The top ten octamer motifs for core, proximal, and distal promoters and all introns for human and Denisovan can be seen in Tables 4 and 5. Information for 5′ and 3’ UTRs are also provided for human.

**Table 4:** Top 10 octamers from different sub-genomic regions in modern human

Motif	Observed	Expected	Score	JASPAR annotation
Core promoters				
GGGGCGGG	4545	19.014	0.996	E2F1
GGGCGGGG	4729	19.014	0.996	
CCCCGCCC	4371	19.014	0.996	EGR1|KLF5|SP1|SP2
GGCGGGGC	3740	19.014	0.995	
GCCCCGCC	3488	19.014	0.995	EGR1|KLF5|SP1|SP2
ggCCCGCCCC	4180	19.014	0.995	EGR1|KLF5|SP1|SP2
GGGGGCGG	2802	19.014	0.993	CTCF
CCGCCCCC	2492	19.014	0.992	EGR1|SP1|SP2
GGCCCCGC	1988	19.014	0.991	
GCGGGGCC	2055	19.014	0.991	
Proximal promoters				
GGGCGGGG	7145	53.738	0.993	
GGGGCGGG	6893	53.738	0.992	E2F1
CCCGCCCC	6585	53.738	0.992	EGR1|KLF5|SP1|SP2
CCCCGCCC	6980	53.738	0.992	EGR1|KLF5|SP1|SP2
GGCGGGGC	5368	53.738	0.99	
GCCCCGCC	5398	53.738	0.99	EGR1|KLF5|SP1|SP2
GGGGGCGG	4443	53.738	0.988	CTCF
CCGCCCCC	4084	53.738	0.987	EGR1|SP1|SP2
CCCCTCCC	4863	79.277	0.984	KLF5|SP1|SP2
GGGAGGGG	4628	79.277	0.983	ZNF263
Distal promoters				
GGGCGGGG	7145	53.738	0.993	
GGGGCGGG	6893	53.738	0.992	E2F1
CCCGCCCC	6585	53.738	0.992	EGR1|KLF5|SP1|SP2
CCCCGCCC	6980	53.738	0.992	EGR1|KLF5|SP1|SP2
GGCGGGGC	5368	53.738	0.99	
GCCCCGCC	5398	53.738	0.99	EGR1|KLF5|SP1|SP2
GGGGGCGG	4443	53.738	0.988	CTCF
CCGCCCCC	4084	53.738	0.987	EGR1|SP1|SP2
CCCCTCCC	4863	79.277	0.984	KLF5|SP1|SP2
GGGAGGGG	4628	79.277	0.983	ZNF263
Introns				
GGGTGGGG	62,500	3466.985	0.947	
GGGGTGGG	59,103	3466.985	0.945	
GGGGAGGG	56,341	3466.985	0.942	ZNF263
GGGCTGGG	55,491	3466.985	0.941	
GGGAGGGG	54,920	3466.985	0.941	ZNF263
CCCCACCC	54,636	3466.985	0.94	KLF5|RREB1
CCCTCCCC	53,123	3466.985	0.939	KLF5|SP1
CCCCTCCC	53,180	3466.985	0.939	KLF5|SP1|SP2
CCCACCCC	51,857	3466.985	0.937	KLF5|RREB1
GGGCAGGG	50,200	3466.985	0.935	
5’ UTR				
GGCGGCGG	3004	21.007	0.993	
GCGGCGGC	3089	21.007	0.993	
GCCGCCGC	2332	21.007	0.991	
CGGCGGCG	2405	21.007	0.991	
CCGCCGCC	2370	21.007	0.991	
GGGGCGGG	1846	21.007	0.989	E2F1
GGGCGGGG	1770	21.007	0.988	
CGCCGCCG	1803	21.007	0.988	
CCCGCCCC	1784	21.007	0.988	EGR1|KLF5|SP1|SP2
CCCCGCCC	1740	21.007	0.988	EGR1|KLF5|SP1|SP2
3’ UTR				
CCCCACCC	4064	143.926	0.966	KLF5|RREB1
CCCACCCC	3940	143.926	0.965	KLF5|RREB1
CCCCTCCC	3774	143.926	0.963	KLF5|SP1|SP2
CCCTCCCC	3679	143.926	0.962	KLF5|SP1
CCCTGCCC	3555	143.926	0.961	ESR1|ESR2|TFAP2A|TFAP2C
CCCAGCCC	3527	143.926	0.961	
GGGTGGGG	3470	143.926	0.96	
GGGGTGGG	3461	143.926	0.96	
GGGAGGGG	3292	143.926	0.958	ZNF263
GGGGAGGG	3207	143.926	0.957	ZNF263

The motif, observed and expected occurrence, the motif score as well as any corresponding annotation from the JASPAR database are all listed for the top 10 motifs from the core, proximal, and distal promoters as well as all introns, and 5′ and 3’ UTRs

**Table 5:** Top 10 octamers from different genomic sub-regions in Denisovan

Motif	Observed	Expected	Score	JASPAR annotation
Core promoters				
GGGGCGGG	17,407	82.475	0.995	EGR1|KLF5|SP1|SP2
GGGCGGGG	17,780	82.475	0.995	
CCCGCCCC	16,718	82.475	0.995	
CCCCGCCC	17,676	82.475	0.995	
GGCGGGGC	13,917	82.475	0.994	EGR1|KLF5|SP1|SP2
GCCCCGCC	13,714	82.475	0.994	
GGGGGCGG	10,283	82.475	0.992	
CCGCCCCC	9785	82.475	0.992	
GGCGGCGG	8520	82.475	0.99	
GCGGGGCG	7806	82.475	0.99	EGR1|SP1|SP2|TFAP2C
Proximal promoters				
GGGCGGGG	26,905	280.231	0.99	
CCCCGCCC	27,739	280.231	0.99	EGR1|KLF5|SP1|SP2
GGGGCGGG	25,911	280.231	0.989	E2F1
CCCGCCCC	26,181	280.231	0.989	EGR1|KLF5|SP1|SP2
GCCCCGCC	20,826	280.231	0.987	EGR1|KLF5|SP1|SP2
GGCGGGGC	19,887	280.231	0.986	
GGGGGCGG	16,398	280.231	0.983	CTCF
CCGCCCCC	15,932	280.231	0.983	EGR1|SP1|SP2
GGCGGCGG	14,897	280.231	0.982	
GCGGCGGC	14,477	280.231	0.981	
Distal promoters				
CAGGCTGG	93,573	1749.587	0.982	
CCAGGCTG	89,626	1749.587	0.981	
CCAGCCTG	88,343	1749.587	0.981	
CAGCCTGG	85,501	1749.587	0.98	
GCCTCCCA	78,433	1749.587	0.978	
GGGCGGGG	35,288	844.888	0.977	
CCCCGCCC	36,139	844.888	0.977	EGR1|KLF5|SP1|SP2
CAGCCTCC	75,312	1749.587	0.977	
GGAGGCTG	71,186	1749.587	0.976	
GCCTGGGC	48,884	1215.815	0.976	NFYB
Introns				
CAGGCTGG	3,328,904	83,571.71	0.976	
CCAGGCTG	3,208,495	83,571.71	0.975	
CCAGCCTG	3,097,321	83,571.71	0.974	
CAGCCTGG	2,984,998	83,571.71	0.973	
GCCTCCCA	2,849,031	83,571.71	0.972	
TTTTTTTT	23,869,680	742,109.9	0.97	
TGGGAGGC	2,675,429	83,571.71	0.97	TAL1::GATA1
CAGCCTCC	2,724,864	83,571.71	0.97	
GGAGGCTG	2,573,566	83,571.71	0.969	
CCTCAGCC	2,528,019	83,571.71	0.968	

The motif, observed and expected occurrence, the motif score as well as any corresponding annotation from the JASPAR database are all listed for the top 10 motifs from the core, proximal, and distal promoters as well as all introns

#### Experimentally verified motifs between human and Denisovan

We searched the scientific literature for examples of genes common to human, Neanderthal, and Denisovan to see whether we could find motifs predicted by our algorithm in any element (e.g., promoters, introns, 5′ or 3’ UTR) of these genes.

#### Top motifs found in human and Denisovan

Among the different sub-genomic regions in human and Denisovan we can see that the top 10 highest-ranking motifs in Tables 4 and 5 match to the well-known E2F1, EGR1, KLF5, SP1, SP2, and ZNF263 motifs.

We found that a variant of the KLF5 binding site (GCCCCGCC) occurs in the promoter of the KLF4 gene. KLF5 is a Krüppel-like transcription factor, which takes part in cell growth, proliferation, and differentiation. Whereas KLF4 inhibits cell growth by interacting with p21 and p53, KLF5 induces cell growth, and its increased mRNA levels can be found in active cells, such as the base of the crypt epithelium as well as the proliferative basal layer of the epidermis, where active cell division takes place in mice. This increase in KLF5 expression is due to the Egr1 protein, which itself is induced by MAPK [27]. Both KLF4 and KLF5 interact with the same cis-element, inhibit each other’s activity, and they also exhibit tumor suppressor and oncogenic activities, respectively.

Frietze et al. [28] studied the distribution of 5000 ZNF263 (a zinc-finger transcription factor) binding sites within the human genome. They found 43 genes that were up-regulated due to ZNF263 and 28 that were down-regulated. We found that the GGGAGGGG cis-element occurs in the promoters of 14 of these genes and that 11 were up-regulated and 3 were down-regulated.

The Ras oncogene is a gene active for example in medullary thyroid cancer [29]. One gene activated by Ras is the calcitonin (CT) gene, the promoter of which contains a Ras-responsive transcriptional element (RREB) at position − 191 - -198 (CCCCCACC), which is a variant of the RREB found in the top ten elements found by our search. This demonstrates that the algorithm is capable of predicting motifs, which have already been experimentally verified.

#### Neanderthal and Denisovan retroviruses in modern humans

So-called endogenous retroviruses (ERVs) make up 5–8% of the human genome. Agoni et al. [30] reported 14 ERVs in the genome sequences of Neanderthal and/or Denisovan fossils. Such elements have also been identified in humans, which also cause disease [31]. Recently, Marchi et al. [32] discovered that eight of the HERVs previously discovered by Agoni et al. were also found in the human genome. These ERVs belong to the HERVK family of endogenous retroviruses, which have been active the most recently, and have seemingly infected the human lineage before modern humans split off Neanderthals and Denisovans. A list of whole genome motifs, which were found in these HERVs are provided in Table 6.

**Table 6:** List of significant whole genome motifs found in Neanderthal and Denisovan ERVs

Motif	HERV id(s)	Score
AGGTGGGA	HERV-K-De1, 3, 6	0.855
CACACCTG	HERV-K-De1, 3, 5, 6	0.889
CAGGTGTG	HERV-K-De2	0.888
GGAGGGGC	HERV-K-De2	0.845
AAAAGAAA	HERV-K-De5, 7	0.862
AAAGAAAA	HERV-K-De5	0.864
TTCTTTCT	HERV-K-De6	0.839
CACACCTGT	HERV-K-De1, 3, 5, 6	0.937
ACAGGTGTG	HERV-K-De2	0.937
CAGGTGTGG	HERV-K-De2	0.916
GTGGAGGGG	HERV-K-De2	0.838
AAGAAAAAG	HERV-K-De5	0.838
AAAAGAAAG	HERV-K-De7	0.848
AAAGAAAGA	HERV-K-De7	0.897
AGAAAAAGA	HERV-K-De5	0.85
AGAAAGAGA	HERV-K-De7	0.834
GAAAAGAAA	HERV-K-De5, 7	0.869
ACACACCTGT	HERV-K-De1, 3, 5, 6	0.873
CACACCTGTG	HERV-K-De1, 3, 5, 6	0.858
CTTTTCCCCA	HERV-K-De1, 3, 5, 6	0.854
CACAGGTGTG	HERV-K-De2	0.853
GGTGTGGAGG	HERV-K-De2	0.86
GTGGAGGGGC	HERV-K-De2	0.849
TGGGGAAAAG	HERV-K-De2, 7	0.852
AAAGAAAGAG	HERV-K-De7	0.882
AAGAAAGAGA	HERV-K-De7	0.879
AGGTGGGA	HERV-K-Ne1	0.855
CACACCTG	HERV-K-Ne1, 2	0.889
TTCTTTCT	HERV-K-Ne1	0.837
CACACCTGT	HERV-K-Ne1, 2	0.937
ACACACCTGT	HERV-K-Ne1, 2	0.873
CACACCTGTG	HERV-K-Ne1, 2	0.858
CTTTTCCCCA	HERV-K-Ne1, 2	0.854

The motif sequence, and the id of the HERV sequence that the motif was found in is given, as well as the motif score

#### Examination of motif content similarity in miR-1304

Besides genes and ERVs, microRNA (miRNA) sequences were examined which were common between human and Denisovan. miRNAs are involved in the regulation of more than 30% of all human genes and take part in complex networks which regulate many cellular processes [12]. There are also a number of miRNAs, which are found only in present-day humans, and are therefore good candidates in discovering differences between modern and archaic humans such as Neanderthal and Denisovan [33]. For example, miR-1304 differs in only one single bp between human and Neanderthal, and is responsible for dental and other craniofacial differences [34].

Overall, ten significant whole genome motifs from human were found in the 22 bp seed sequence of miR-1304 (CACATCTCACTGTAGCCTC[A/G]AA), which are listed in Table 6. MiR-1304 has also been shown to regulate two genes, ENAM and AMTN, which code for the enamelin and amelotin proteins, which take part in odontogenesis [35]. Statistically significant motifs were also found which occur in the 3’ UTR of these genes (Table 7).

**Table 7:** Whole genome motifs in miR-1304 and 5′ and 3’ UTR of the ENAM and AMTN genes

Motif	Target	Score
Human		
CCCTGC	ENAM 3’ UTR	0.845
TCCCTGC	ENAM 3’ UTR	0.836
TTTCCTTTT	ENAM 3’ UTR	0.819
GCTGCC	AMTN 3’ UTR	0.816
GCTGCCT	AMTN 3’ UTR	0.835
Neanderthal		
ACTGTAGCCT	miR-1304 seed	0.887
CACTGTAGCC	miR-1304 seed	0.85
AAAAAA	ENAM 3’ UTR	0.84
CCTGCC	ENAM 3’ UTR	0.821
CCCTGCC	ENAM 3’ UTR	0.837
CCTGCCT	ENAM 3’ UTR	0.891
CTGCCTC	ENAM 3’ UTR	0.898
AATCACTTG	ENAM 3’ UTR	0.846
CCTGCCTCG	ENAM 3’ UTR	0.92
TTTCCTTTT	ENAM 3’ UTR	0.866
TTTTTT	AMTN 3’ UTR	0.84
CTGCCTC	AMTN 3’ UTR	0.898

Several statistically significant whole genome motifs from modern human and Neanderthal were found in the seed section of the miR-1304 miRNA, as well as the 3′ and 5’ UTR of the ENAM and AMTN genes

#### Human accelerated regions

Until now, many molecular genetic studies have focused on analyzing the coding sequences of genes, which are different between humans and other species. However, since many non-coding genetic elements make up the majority of functional elements in the genome, it would certainly be worthwhile to investigate these regions in order to identify such elements and to see what kinds of possible differences there are between human, Neanderthal and Denisovan [6, 23].

We found that the statistically significant decamer GCAGCCTTGG was found in intron 9 of the CENTG2 gene in both human (score = 0.867) and Denisovan [7]. This gene is responsible for differential limb development patterns. This intron contains a 546-bp region called HACNS1, or Human Accelerated Non-Coding Sequence 1, but is constrained in all but 16 human-specific positions between human, chimp, rhesus, mouse, rat, dog, chicken, and frog. A shorter, 81-bp segment contains 13 of these 16 bp differences. This 81-bp long segment is identical in both human and Denisovan. The hexamer CAGCCT (score = 0.842), and the nonamers GCAGCCTTG (score = 0.857) and GGCACCCAC (score = 0.843) were also among the top motifs that the algorithm found in Denisovan.

Also, Pollard et al. [23] identified 49 so-called Human Accelerated Regions (labelled HAR1–49) in the human genome that had substantial sequence differences compared to other animals. 94% (46 of 49) of these are located in non-coding regions; 26 are found in intergenic regions, 20 in introns, 2 in coding regions, and 1 in RNA.

Each of the 26 intergenic regions has a BLAST hit with both the Neanderthal and the Denisovan genome, and the 20 intronic regions also have a BLAST hit with the Denisovan genome. Significant motifs from the human, Neanderthal, and Denisovan genomes and the human and Denisovan intronic regions are listed in Additional file 5, along with the HAR region that they map to.

Since a large number of base pair differences are present within these elements compared to other mammals (for example there is an 18 bp difference between human and chimpanzee in HAR1), it would be interesting to see how much of a difference exists between modern humans, Neanderthals, and Denisovans.

Between modern humans, Neanderthal and Denisovan, there are 27 whole genome motifs which map to these intergenic regions. There are 66 motifs unique to modern human, 4 to Neanderthal, and 127 which are unique to both Neanderthal and Denisovan. The number of motifs common to different combinations of subspecies can be seen in Fig. 4. The four whole genome motifs unique to Neanderthal are CTTTGGGA, AGAAAATGTG, AAGTGCTG and ACAGGCTCTG.

Between humans and Denisovans there are 82 motifs which are unique to human introns. These motifs can be seen in Additional file 6. The number of motifs unique to modern human or Denisovan, and which are common to both can be seen in Fig. 5. We searched the human intron sequence set for these specific human-specific motifs, to see what kind of genes they fall in. The top 20 genes which have at least 77 unique motifs are listed in Table 8 along with their gene name and function.

**Table 8:** Top 20 human genes with highest number of human-specific intronic motifs from analysis of HAR regions

Refseq ID	Gene name	Function	Number of motifs
NP_955533.1	PRDM16	PR domain containing 16 isoform 2	81
NP_689957.3	SDK1	protein sidekick-1 isoform 1	81
NP_570858.2	PTPRN2	receptor-type tyrosine-protein phosphatase N2 isoform 3	81
NP_570857.2	PTPRN2	receptor-type tyrosine-protein phosphatase N2 isoform 2	81
NP_115821.2	MEGF11	multiple epidermal growth factor-like domains protein 11 precursor	80
NP_113654.3	TRAPPC9	trafficking protein particle complex 9	80
NP_071407.4	CDH23	cadherin-23 isoform 1 precursor	79
NP_071397.2	PRDM16	PR domain containing 16 isoform 1	79
NP_065828.2	SORCS2	VPS10 domain-containing receptor SorCS2 precursor	79
NP_060021.1	GRID1	glutamate receptor ionotropic, delta-1 precursor	78
NP_056030.1	CAMTA1	calmodulin-binding transcription activator 1 isoform 1	78
NP_055729.2	AGAP1	arf-GAP with GTPase, ANK repeat and PH domain-containing protein 1 isoform 2	77
NP_003362.2	VAV2	guanine nucleotide exchange factor VAV2 isoform 2	77
NP_002838.2	PTPRN2	receptor-type tyrosine-protein phosphatase N2 isoform 1 precursor	77
NP_001785.2	CDH4	cadherin-4 isoform 1 preproprotein	77
NP_001127870.1	VAV2	guanine nucleotide exchange factor VAV2 isoform 1	77
NP_001104488.1	NAV2	neuron navigator 2 isoform 3	77
NP_001085.2	ASIC2	acid-sensing ion channel 2 isoform MDEG1	77
NP_001076044.1	RBFOX3	RNA binding protein fox-1 homolog 3	77
NP_001032208.1	AGAP1	arf-GAP with GTPase, ANK repeat and PH domain-containing protein 1 isoform 1	77

Statistically significant intron motifs were found in 20 intronic HARs between modern human and Denisovan. Eighty-two of them were shown to be specific to modern human. These are the top 20 genes with the highest number of these human-specific intron motifs (at least 77 of them) in their introns. Listed are the genes’ Refseq ID, their gene name, function and the number of motifs their introns contain

Furthermore, beyond the top 20 genes, we took the top 189 transcript IDs which had the highest number of motifs (at least 71 unique motifs) coming from the 82 unique human intron motifs examined previously. These 189 transcripts subsequently mapped to 135 UniProt IDs at the UniProt website, which in turn mapped to 261 specific gene names. GO term analysis was done with these 261 genes (*p* < 0.05) at the Panther Database website, the result of which can be seen in Tables 9 and 10. The results of the GO term analysis are also available in Additional file 7.

**Table 9:** Biological processes found through GO term analysis

GO term	Genes in database	Genes found	Expected	Fold enrichment	*P*-value
Single-organism developmental process	5316	61	27.63	2.21	8.94E-08
Developmental process	5402	61	28.08	2.17	1.84E-07
System development	4138	52	21.51	2.42	3.96E-07
Single-multicellular organism process	5509	61	28.63	2.13	4.41E-07
Anatomical structure development	5059	58	26.29	2.21	5.19E-07
Cell adhesion	1103	25	5.73	4.36	3.16E-06
Biological adhesion	1108	25	5.76	4.34	3.47E-06
Multicellular organism development	4733	54	24.6	2.2	5.41E-06
Single-organism process	12,622	94	65.6	1.43	2.04E-05
Multicellular organismal process	6584	64	34.22	1.87	3.08E-05
Nervous system development	2200	33	11.43	2.89	1.04E-04
Cell-cell adhesion	680	18	3.53	5.09	1.27E-04
Cellular developmental process	3501	42	18.2	2.31	3.60E-04
Cell differentiation	3425	41	17.8	2.3	6.09E-04
Regulation of multicellular organismal process	2656	35	13.8	2.54	8.50E-04
Homophilic cell adhesion via plasma membrane adhesion molecules	155	9	0.81	11.17	1.18E-03
Generation of neurons	1391	24	7.23	3.32	1.37E-03
Cell-cell adhesion via plasma-membrane adhesion molecules	212	10	1.1	9.08	1.57E-03
Regulation of cellular component organization	2292	31	11.91	2.6	3.40E-03
Neurogenesis	1487	24	7.73	3.11	4.57E-03
Regulation of developmental process	2263	30	11.76	2.55	8.48E-03
Neuron differentiation	937	18	4.87	3.7	1.38E-02
Cell development	1476	23	7.67	3	1.50E-02
Regulation of biological quality	3560	39	18.5	2.11	1.51E-02
Regulation of multicellular organismal development	1714	25	8.91	2.81	1.60E-02
Anatomical structure morphogenesis	1959	27	10.18	2.65	1.66E-02
Single organism signaling	5262	50	27.35	1.83	1.69E-02
Signaling	5266	50	27.37	1.83	1.73E-02
Regulation of cell projection organization	586	14	3.05	4.6	1.92E-02
Single-organism cellular process	9804	75	50.96	1.47	2.22E-02
Regulation of nervous system development	782	16	4.06	3.94	2.52E-02
Multicellular organismal signaling	129	7	0.67	10.44	4.71E-02

The top 189 transcripts, which had the highest number of human-specific intron motifs mapped to 135 UniProt IDs, which also mapped to 261 specific gene names. GO term analysis was performed with these genes at the Panther website. Shown below are the top 32 biological process GO terms found in this GO analysis

**Table 10:** Cellular components found through GO term analysis

GO term	Genes in database	Genes found	Expected	Fold enrichment	*P*-value
Synapse	793	21	4.12	5.1	1.05E-06
Cell periphery	5394	56	28.03	2	1.20E-05
Cell junction	1374	25	7.14	3.5	4.00E-05
Synapse part	656	17	3.41	4.99	7.04E-05
Neuron part	1310	24	6.81	3.52	7.04E-05
Plasma membrane	5285	53	27.47	1.93	1.51E-04
Cation channel complex	207	10	1.08	9.29	1.97E-04
Ion channel complex	283	11	1.47	7.48	3.93E-04
Transmembrane transporter complex	321	11	1.67	6.59	1.34E-03
Plasma membrane part	2671	33	13.88	2.38	1.55E-03
Transporter complex	328	11	1.7	6.45	1.64E-03
Postsynapse	402	12	2.09	5.74	1.76E-03
Neuron projection	974	18	5.06	3.56	3.69E-03
Cell projection	1862	25	9.68	2.58	1.08E-02
Potassium channel complex	92	6	0.48	12.55	1.25E-02

Shown below are the top 15 cellular component GO terms found in the GO analysis at the Panther database

#### Conserved deleted regions in human

McLean et al. [36] studied 583 so-called hCONDEL sequences in chimpanzee and human. These are called hCONDELs because they appear in highly conserved intergenic regions of the genome, and are present in chimpanzee, yet missing in human. Five hundred ten of these regions were validated experimentally by single reads, which span both sides of the region in the human genome. These hCONDEL regions have a median size of 2804 bp and show a skew towards GC-poor regions. These regions also fall close to genes, which take part in steroid hormone receptor signaling. Since these regions fall within intergenic regions, they might contain regulatory elements in the chimpanzee genome, which might cause phenotypic differences as compared to the human genomes.

We thought that it would be interesting to see whether these hCONDEL regions were also fully missing from the Neanderthal and Denisovan genomes. If some of them are also present in these two genomes, they might shed light on to possible differences between modern and archaic humans.

Therefore, after having extracted the hCONDEL regions from the *Pan troglodytes* genome, version 2, we blasted them against the Neanderthal and Denisovan genomes. Of the 583 hCONDEL regions, 287 (49.2%) of them had a significant hit at least 50 bp long, with at least 90% sequence identity.

We then looked to see how many significant genomic Denisovan and Neanderthal motifs (lengths 6 to 10) fall into these 287 dCONDEL and nCONDEL regions. These numbers are summed up in Table 11. As we can see, there is quite a large overlap between Neanderthal and Denisovan. A list of these motifs for both Neanderthal and Denisovan for motif lengths 6 to 10 can be found in Additional file 8.

**Table 11:** Number of significant genome motifs in nCONDEL and dCONDEL regions

	Hexamers	Heptamers	Octamers	Nonamers	Decamers
Neanderthal	24	107	627	2206	6365
Denisovan	24	107	627	2195	6356
difference	0	0	6	29	73
Only in Denisovan	0	0	3	9	32
Only in Neanderthal	0	0	3	20	41

Five hundred eighty-three hCONDEL regions present in chimpanzee were BLASTED against the Neanderthal and Denisovan genomes, for which there were 287 nCONDELs and dCONDELs. The number of motifs (length 6–10 bp) present in Neanderthal and Denisovan are given, as well as the number of motifs present in both species or unique to either Neanderthal or Denisovan

## Discussion

We have performed the motif content analysis of the human, Neanderthal and Denisovan genome. With this analysis, we provide a catalogue of motifs and their motif score in seven and five sub-genomic regions in the human and Denisovan genome as well as the Neanderthal whole genome. This data is now available for other researchers to use and analyze further.

One of the main questions in this analysis is whether our predicted motifs have actual biological relevance. As we can see, for the three different types of promoter sets, the intron sets, and the 5′ and 3’ UTR sequence sets, the highest-ranking motifs matched experimentally verified motifs which had already been described in the JASPAR database. Furthermore, they did so in a highly non-random manner. When comparing the predicted motifs based on their match with experimentally verified motifs, the experimentally verified ones have higher ranks, according to *p*-value measurements.

However, as a further test we wanted to see if the statistically significant motifs that we predicted fall within biologically active sites within the genome. We found that a number of our candidate motifs fall within the sequence of a number of HERV sequences, and the miRNA sequence miR-1304 and also fall within the 3’ UTRs of a couple of genes which are regulated by this latter miRNA.

Another interesting area of validation was comparing regions which were either different in sequence between human and other species, or were missing from humans compared to Denisovan and Neanderthal. These were the HAR regions as well as the hCONDEL regions.

The study of over-represented statistically significant genome motifs in the 49 HAR regions [23] also validated the biological validity of our predicted motifs. Some of the top 20 genes found in this search are for example, neuron navigator 2 isoform 3 (NAV2), which is required for all-trans retinoic acid to induce neurite outgrowth in human neuroblastoma cells [37]. Another gene is SorCS2, which is a VPS10-domain family receptor, which takes part in protein trafficking, intracellular and intercellular signaling [38]. SorCS2 itself is expressed in the hippocampus and is also involved in synapse formation and neuron function [39]. Another gene, TRAPPC9 (trafficking protein particle complex 9) is highly expressed in the post-mitotic neurons of the cerebral cortex, and mutations in this gene show defects in axonal connectivity [40]. GRID1, which encodes the glutamate D1 receptor, which is a member of the δ-family of ionotropic glutamate receptors, acts like an adhesion molecule by linking the postsynaptic and presynaptic compartments [41]. Yet another gene, PRDM16 is responsible for regulating the amount of mitochondrial reactive oxygen species (mtROS), which is necessary for the development of neurons [42]. The deletion of CAMTA1 causes cerebellar atrophy and Purkinje cell degeneration in mice [43]. The acid-sensing ion channels (ASICs) form mechanoreceptors in the periphery, and localize to dorsal and lumbar root ganglia [44]. It is highly interesting that a number of genes with high motif content were found in this search, since Pollard et al. [23] found that 24% of the HAR regions they described were adjacent to neurodevelopmental genes. This validates the fact that the motifs that we found are indeed biologically relevant and meaningful.

As we can see, five of the top 32 biological function GO terms, and five of the top 15 cellular component GO terms are involved in neurogenesis and neuron development, which are indicative of the differential neurological functions between modern and archaic humans. Not only that, but GO terms for ion channel complexes and transmembrane transporter complexes were also found. No significant GO terms for molecular processes were retrieved.

Analyzing hCONDEL regions also produced interesting results. These regions are specifically missing from the human genome, and as such gave us an opportunity to analyze the motif content difference between Neanderthal and Denisovan. Overall, it seems that the whole genome sequence similarity between these two archaic human subspecies is very high, since they both contained the same 287 hCONDEL regions, which are missing from human.

Overall, 44 statistically significant whole genome motifs mapped to these 287 regions, which differ between Neanderthal and Denisovan. Of these, among the motifs which only occur in Denisovan, the motif TGCCCAGACT (score = 0.862) corresponds to the P63 Responsive Element [45]. P63, a member of the TP53 family of proteins is involved in certain types of tumors, such as vulvar cancer. Its expression is negatively correlated by miR-223-5p [46]. Among the 64 statistically significant whole genome motifs, which differ between Neanderthal and Denisovan, and which only occur in Neanderthal, the nonamer AGAGGGAG corresponds to the SP2 motif, and the CCAGGCCT motif corresponds to the TP63 motif identified earlier.

## Conclusions

In summary, we have seen that despite Neanderthal and Denisovan having gone extinct, we are still able to discern certain genetic elements, which shed light onto the possible phenotypic differences between the two archaic human subspecies as well as modern human. Indeed, it would also be highly interesting to see what similarities and differences are there between these three subspecies and other fossil hominids.

In Table 2 we can see that the CG% between Neanderthal and Denisovan are almost identical (at most there is a 0.01% difference), whereas the CG% between modern human and the two archaic human subspecies is 0.25–0.26%. However, the variation of GC% between any two modern human individuals can even exceed this level of variation. For example, Merchant et al. [47] estimated the GC% of human to be 41%, whereas we have 40.4%. Furthermore, when we ran chi-squared analysis on the GC% of the three human subspecies, we found that the *p*-values pertaining to each chi-square statistic were all statistically insignificant, given the null hypothesis that the GC% of all three subspecies come from the same distribution. Therefore, we do not believe that these differences in GC% between modern and archaic humans is statistically significant.

In order to get a picture of the biological differences between the motif distribution between the three genomes, we looked at the set of statistically significant motifs which were unique to modern human. We could only do this because gene annotation is available only for modern human, the other two subspecies being extinct. This was done separately for motifs of lengths 6–10, for core, proximal, and distal promoters as well as introns between modern humans and Denisovan. This is because of the two archaic human subspecies, only Denisovan had these genomic subregions available. These sets of statistically significant, modern human-unique motifs can be found in Additional file 9 (shown in the first four tabs for core, proximal and distal promoters). Also available in this file is the number of such motifs which were found in the top 100 (core, proximal, distal) promoter/intron region of the genes in modern human (Tables 5, 6, 7 and 8). In the “top genes (based on promoters)” tab of Additional file 9 we can see a list of 50 individual genes variants for core, proximal and distal promoters, which came from at least three of the top 100 lists mentioned previously.

These correspond to 38 individual genes, which are listed in Table 12, along with their annotation. The motif repertoire found in core promoters might be different from that of proximal and distal promoters because core promoter motifs take part in the active transcription of genes, whereas proximal/distal promoters play a more modulatory/regulatory role. This is because general transcription factors are found within the core promoters (therefore not too many genes were found with human-specific motifs), whereas the proximal and distal promoters contain more specific regulatory motifs, unique to modern humans. When these 38 genes were entered into the GeneOntology database, two statistically significant (FDR < 5%) GO terms came up: regulation of histone modification (GO:0031056), and animal organ development (GO:GO:0048513).

**Table 12:** List of 38 human genes with a high number of human-unique motifs found in proximal and distal promoters

Gene symbol	GeneCard Annotation
ANKRD11	Ankyrin Repeat Domain 11
ANO9	Anoctamin 9
C1orf170	PPARGC1 And ESRR Induced Regulator, Muscle 1
CKB	Creatine Kinase B
CUX1	Cut Like Homeobox 1
DVL1	Dishevelled Segment Polarity Protein 1
EEF1D	Eukaryotic Translation Elongation Factor 1 Delta
FGFRL1	Fibroblast Growth Factor Receptor Like 1
GNAQ	G Protein Subunit Alpha Q
IGF1R	Insulin Like Growth Factor 1 Receptor
IGF2	Insulin Like Growth Factor 2
ING2	Inhibitor Of Growth Family Member 2
KIF1A	Kinesin Family Member 1A
KLF16	Kruppel Like Factor 16
LIMD2	LIM Domain Containing 2
MTA1	Metastasis Associated 1
NOC2L	NOC2 Like Nucleolar Associated Transcriptional Repressor
NSD1	Nuclear Receptor Binding SET Domain Protein 1
POLE	DNA Polymerase Epsilon, Catalytic Subunit
PQLC1	PQ Loop Repeat Containing 1
PWWP2B	PWWP Domain Containing 2B
RAC3	Rac Family Small GTPase 3
RASA3	RAS P21 Protein Activator 3
RFNG	RFNG O-Fucosylpeptide 3-Beta-N-Acetylglucosaminyltransferase
RUNX1	Runt Related Transcription Factor 1
SAMD11	Sterile Alpha Motif Domain Containing 11
SAMD4A	Sterile Alpha Motif Domain Containing 4A
SDC1	Syndecan 1
SEMA4C	Semaphorin 4C
SKI	SKI Proto-Oncogene
SLC19A1	Solute Carrier Family 19 Member 1
SLC2A4RG	SLC2A4 Regulator
SPHK1	Sphingosine Kinase 1
STK11	Serine/Threonine Kinase 11
SUV420H1	Lysine Methyltransferase 5B
TRIM8	Tripartite Motif Containing 8
TSEN54	TRNA Splicing Endonuclease Subunit 54
UBTF	Upstream Binding Transcription Factor, RNA Polymerase I

Statistically significant human-specific motif lists (lengths 6–10) were determined for core, proximal and distal promoters. These motifs were searched for in the appropriate human promoter set. The top 100 genes with the highest number of such motifs in their promoters were listed. Those 38 genes listed below belonged to at least three top 100 lists, and were found to be in common between proximal and distal promoters

The human-unique intron motifs (lengths 6–10) were also searched for in the human intron sequence set, and the top 100 genes were selected with the highest number of human-unique motifs. These are also listed in Additional file 9. The tab “top genes (based on introns)” shows those 104 gene variants (corresponding to 59 genes), which were listed in at least three out of five top 100 gene lists. These 59 genes were searched for at the Gene Ontology website, and were shown to be associated with 33 GO terms, listed in Table 13. What is interesting is that 11 of the 33 GO terms were shown to be involved with neural activity: neuron projection development and morphogenesis, neuron development, neurogenesis, generation of neurons, neuron differentiation, nervous system development, and related terms, such as vocalization, sensory organ development, and neuromuscular processes controlling balance, and neuromuscular processes. GO terms for exon development and axonogenesis were also found. For cellular components, these 59 genes were associated with two other statistically significant GO terms, namely synapse (GO:0045202) and neuron part (GO:0097458). These results correlate well with what we found in the analysis of Human Accelarated Regions, and even found that 11 of these top 59 genes match genes from the analysis of HAR regions (see Table 8). These genes found in modern human compared to Denisovan imply that the regulatory motifs found in them are responsible for differential development in neural functions, possibly cognitive abilities, as well as sensory perception and vocalization. These differences may have accrued after the divergence between modern and archaic humans.

**Table 13:** GO terms (biological processes) found for the 59 genes with a high number of human-unique intron motifs

GO term	GO term id	No. genes	FDR
System development	GO:0048731	37	1.39E-08
Multicellular organism development	GO:0007275	38	7.48E-08
Anatomical structure development	GO:0048856	39	9.04E-08
Developmental process	GO:0032502	40	1.06E-07
Multicellular organismal process	GO:0032501	43	3.78E-07
Nervous system development	GO:0007399	25	7.65E-07
Cell-cell adhesion via plasma-membrane adhesion molecules	GO:0098742	9	3.47E-05
Cell-cell adhesion	GO:0098609	11	8.49E-05
Animal organ development	GO:0048513	25	2.47E-04
Neuron differentiation	GO:0030182	14	3.44E-04
Homophilic cell adhesion via plasma membrane adhesion molecules	GO:0007156	7	5.16E-04
Biological adhesion	GO:0022610	13	9.89E-04
Cell adhesion	GO:0007155	13	1.01E-03
Cellular developmental process	GO:0048869	26	1.19E-03
Neurogenesis	GO:0022008	16	2.13E-03
Cell differentiation	GO:0030154	25	2.70E-03
Generation of neurons	GO:0048699	15	4.06E-03
Anatomical structure morphogenesis	GO:0009653	18	5.49E-03
Neuron development	GO:0048666	11	5.75E-03
Neuromuscular process	GO:0050905	5	1.04E-02
Sensory organ development	GO:0007423	9	1.10E-02
Neuromuscular process controlling balance	GO:0050885	4	1.37E-02
Vocalization behavior	GO:0071625	3	2.08E-02
Cell projection morphogenesis	GO:0048858	8	2.63E-02
Neuron projection development	GO:0031175	9	2.64E-02
Cell development	GO:0048468	14	2.70E-02
Retina layer formation	GO:0010842	3	2.73E-02
Neuron projection morphogenesis	GO:0048812	8	2.76E-02
Plasma membrane bounded cell projection morphogenesis	GO:0120039	8	2.81E-02
Axonogenesis	GO:0007409	7	3.04E-02
Cell part morphogenesis	GO:0032990	8	3.22E-02
Cell morphogenesis	GO:0000902	9	3.59E-02
Axon development	GO:0061564	7	4.66E-02

Statistically significant human-specific motif lists (lengths 6–10) were determined for introns. These motifs were searched for in the human intron sequence set. The top 100 genes with the highest number of such motifs in their introns were listed. Those 59 genes were listed which belonged to at least 3 top 100 lists, and plugged into the Gene Ontology database. Below are listed 33 GO terms associated with these 59 genes

Since the sequencing of the whole genome was made possible for Neanderthal and Denisovan [1–3], despite the degraded quality of DNA, it surely might be possible to sequence the genomes of such fossil hominin species such as *Homo erectus*, *Homo habilis*, or the newly discovered *Homo naledi* [48], for example. With the whole genome sequence of a larger number of fossil hominins available, we would be able to make large-scale genomic analyses and comparisons possible. Even though we do not have access to gene expression or protein data, we can still learn a lot from comparing the genome motif content between different modern and archaic human subspecies.

## Additional files

Additional file 1: List of statistically significant motifs for modern human. The motif sequence, number of observed and expected occurrences as well as motif score are listed for motif lengths 6–10 in the whole genome, core, proximal, and distal promoters, as well as all introns, 5′ and 3’ UTRS, as well as average score and standard deviation for all motif lengths and genomic sub-regions. (XLSX 219664 kb)

Additional file 2: List of statistically significant motifs for Neanderthal. The motif sequence, number of observed and expected occurrences as well as motif score are listed for motif lengths 6–10 in the whole genome, as well as average score and standard deviation for all motif lengths. (XLSX 43083 kb)

Additional file 3: List of statistically significant motifs for Denisovan. The motif sequence, number of observed and expected occurrences as well as motif score are listed for motif lengths 6–10 in the whole genome, core, proximal, and distal promoters, as well as all introns, as well as average score and standard deviation for all motif lengths and genomic sub-regions. (XLSX 191122 kb)

Additional file 4: List of statistically significant motifs for mouse. The motif sequence, number of observed and expected occurrences as well as motif score are listed for motif lengths 6–10 in the whole genome, core, proximal, and distal promoters, as well as all introns, 5′ and 3’ UTRS, as well as average score and standard deviation for all motif lengths and genomic sub-regions. (XLSX 247147 kb)

Additional file 5: *P*-values for common motifs between modern modern human and Denisovan. P-values were calculated for common motifs for the whole genome, core, proximal, and distal promoters as well as all introns according to the hypergeometric distribution for lengths 6–10 bp. The length of the motif, the number of possible motifs as well as the number of common motifs, as well as the *p*-value is listed. (PDF 319 kb)

Additional file 6: List of statistically significant motifs in 49 HAR regions studied by Pollard et al., 2006 [23]. List of statistically significant genomic motifs found in 26 HARs in modern human, Neanderthal and Denisovan and in 20 intronic HARs in modern human, Neanderthal and Denisovan, as well as a list of the 82 intronic motifs unique to modern humans. (XLSX 36 kb)

Additional file 7: Results of GO analysis. A list of 82 statistically significant intron motifs from 20 intron HARs unique to human is provided. The list of 135 Uniprot IDs is listed as well as the 261 human genes that they correspond to. The results of the GO analysis for biological processes and cellular components are listed. (XLSX 27 kb)

Additional file 8: Intergenic motifs in nCONDELs and dCONDELs. List of statistically significant motifs in nCONDELS and dCONDELs in Neanderthal and Denisovan. These are statistically significant whole genome motifs which were found in regions of the Neanderthal and Denisovan genomes which are missing from the genome of modern human. (XLSX 191 kb)

Additional file 9: List of human specific motifs for motif lengths 6–10 from core, proximal, distal promoters and introns as compared to Denisovan. Statistically significant motifs of lengths 6–10 from core, proximal, and distal promoters as well as introns unique to modern humans are listed (and not found in Denisovan). These motifs were found in the corresponding genomic subregion in modern human, and the number of such human-unique motifs were listed for each gene variant. From these lists the top 100 genes were selected which had the highest number of human-unique motifs in their promoter/intron. Listed in tab “top genes (based on promoters)” are genes which were found in at least three top 100 lists. This was done separately for core, proximal, and distal promoters, and also for introns in the tab “top genes (based on introns)”. Fifty gene variants (38 genes) were found which both in the proximal and distal promoter sets appeared. One hundred four genes (their NP id, GeneCard name and annotation) were found which were listed in at least 3 top 100 gene lists whose introns contained statistically significant human-unique intron motifs. (XLSX 5181 kb)

## Abbreviations

AMTN: Amelotin
ASIC: Acid-sensing ion channel
CAMTA1: Calmodulin-binding transcription activator 1 isoform 1
CENTG2: AGAP1 (ArfGAP with GTPase domain, ankyrin repeat and PH domain 1)
CONDEL: CONserved DELetion
E2F1: E2F transcription factor 1
EGR1: Early growth response 1 factor
ENAM: Enamelin
EPD: European Promoter Database
ERV: Endogenous Retroviros
GRID1: Glutamate receptor ionotropic, delta-1 precursor
HACNS: Human accelerated conserved non-coding sequences
HAR: Human accelerated regions
HERV: Human Endogenous Retroviros
HND: Human, Neanderthal, Denisovan
KLF4: Kruppel-like factor 4
KLF5: Kruppel-like factor 5
MAPK: Mitogen activated kinase-like protein
NAV2: Neuron navigator 2
P53: Tumor protein 53
P63: Tumor protein 63
PFM: Position frequency matrix
PRDM16: PR domain containing 16 isoform 1
PWM: Position weight matrix
RREB: Ras Responsive Element Binding Protein
SorCS2: VPS10 domain-containing receptor SorCS2 precursor
SP1: Sp1 transcription factor
SP2: Sp2 transcription factor
TRAPPC9: Trafficking protein particle complex 9
ZNF263: Zinc finger protein 263
