# Supplementary material for: Motifome comparison between modern human, Neanderthal and Denisovan
Source: BMC Genomics. 2018 Jun 18;19:472. doi: 10.1186/s12864-018-4710-1 (PMC6006668; doi:10.1186/s12864-018-4710-1)
Supplement: Supplementary file 5 — P-values for common motifs between modern modern human and Denisovan. P-values were calculated for common motifs for the whole genome, core, proximal, and distal promoters as well as all introns according to the hypergeometric distribution for lengths 6–10 bp. The length of the motif, the number of possible motifs as well as the number of common motifs, as well as the p-value is listed. (PDF 319 kb) [file 12864_2018_4710_MOESM5_ESM.pdf]

motif length	number of possible motifs	top motifs, modern human	top motifs, Denisova	common motifs	p_value
whole genome					
6	4096	1000	1000	862	0
7	16384	1000	1000	702	0
8	65536	1000	1000	453	0
9	262144	1000	1000	172	1E-226
10	1048576	1000	1000	52	3E-71
core promoters					
6	4096	1000	1000	965	0
7	16384	1000	1000	938	0
8	65536	1000	1000	907	0
9	262144	1000	1000	905	0
10	1048576	1000	1000	869	0
proximal promoters					
6	4096	1000	1000	913	0
7	16384	1000	1000	864	0
8	65536	1000	1000	789	0
9	262144	1000	1000	719	0
10	1048576	1000	1000	631	0
distal promoters					
6	4096	1000	1000	836	0
7	16384	1000	1000	733	0
8	65536	1000	1000	589	0
9	262144	1000	1000	348	0
10	1048576	1000	1000	189	0
all introns					
6	4096	1000	1000	849	0
7	16384	1000	1000	664	0
8	65536	1000	1000	411	0
9	262144	1000	1000	148	3E-184
10	1048576	1000	1000	39	7.1E-49
